# Circadian clock proteins control adaptation to novel environment and memory formation

**DOI:** 10.18632/aging.100142

**Published:** 2010-05-02

**Authors:** Anna A.Kondratova, Yuliya V.Dubrovsky, Marina P. Antoch, Roman V. Kondratov

**Affiliations:** ^1^ Lerner Research Institute, Cleveland Clinic, Cleveland, OH 44195, USA; ^2^ Department of Biological, Geological and Environmental Sciences and Center for Gene Regulation in Health and Disease, Cleveland State University, Cleveland, OH 44115, USA; ^3^ Department of Molecular and Cellular Biology, Roswell Park Cancer Institute, Buffalo, NY 14203, USA

**Keywords:** transcription, aging, oxidative stress, hippocampus, gene expression

## Abstract

Deficiency of the transcription factor BMAL1, a core component of the circadian clock, results in an accelerated aging phenotype in mice. The circadian clock regulates many physiological processes and was recently implicated in control of brain-based activities, such as memory formation and the regulation of emotions. Aging is accompanied by the decline in brain physiology, particularly decline in the response and adaptation to novelty. We investigated the role of the circadian clock in exploratory behavior and habituation to novelty using the open field paradigm. We found that mice with a deficiency of the circadian transcription factor BMAL1 display hyperactivity in novel environments and impaired intra- and intersession habituation, indicative of defects in short- and long-term memory formation. In contrast, mice double-deficient for the circadian proteins CRY1 and CRY2 (repressors of the BMAL1-mediated transcription) demonstrate reduced activity and accelerated habituation when compared to wild type mice. Mice with mutation in theClock gene (encoding the BMAL1 transcription partner) show normal locomotion, but increased rearing activity and impaired intersession habituation. BMAL1 is highly expressed in the neurons of the hippocampus - a brain region associated with spatial memory formation; BMAL1 deficiency disrupts circadian oscillation in gene expression and reactive oxygen species homeostasis in the brain, which may be among the possible mechanisms involved. Thus, we suggest that the BMAL1:CLOCK activity is critical for the proper exploratory and habituation behavior, and that the circadian clock prepares organism for a new round of everyday activities through optimization of behavioral learning.

## Introduction

The ability to explore the outside world and compare old and new information is critical for animal's survival. The proper exploratory behavior in novel unpredictable situations (brought about by weather changes, activity of other animals, etc.) allows distinguishing meaningful and ignoring not important novel stimuli and their combinations. Habituation, one of the simplest forms of non-associative learning, is the mechanism providing an animal with the means to dampen the perception of repetitive neutral stimuli and be ready to effectively detect a novel stimulus with a yet unknown significance, and therefore is vitally important for the interaction of an organism with its environment [1-3]. Both response to novelty and habituation change with age, but molecular mechanisms underlying these changes remain mostly unknown.

Recently we have demonstrated that a deficiency of the transcription factor BMAL1 in mice results in accelerated aging [4]. BMAL1 activity is critical for the operation of the circadian clock - a genetically determined time-keeping system generating 24-hour oscillations in physiology and behavior known as the circadian rhythms [5]. The involvement of the circadian clock in the control of brain-based activities such as sleep [6], reward behavior [7,8] and regulation of mood [9,10] has been reported. Recently, a connection between the circadian clock and memory has been suggested: mice with deficiencies of different components of the circadian clock demonstrate impairments of some types of memory and learning [11]. Here we hypothesize that the circadian clock is involved in the regulation of the adaptation to the new environment, and investigate this hypothesis using a set of circadian mutants - mice with targeted disruptions of circadian genes *Bmal1* or *Cry1 *and *Cry2*, or with the mutation of the *Clock* [12-14] gene. These genes encode proteins representing the core components of the circadian clock. Transcription factors BMAL1 and CLOCK form a transcription complex activating expression of target genes including circadian transcription repressors CRY1 and CRY2. In turn, CRY1 and CRY2 suppress activity of the BMAL1:CLOCK complex, including their own expression, thus generating a negative feedback loop; expression of several other genes important for the functional clock (i.e. *Per1, 2 and 3*) is also under the transcription control of the BMAL1:CLOCK complex [15].

## Results

### Hyperactivity and impaired habituation of *Bmal1-/-* mice

**Figure 1.**
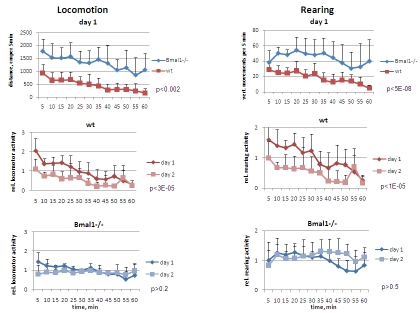
Open field analysis of exploratory activity and habituation of wild type and *Bmal1-/-* mice. Locomotor and rearing activity were measured in 5 min increments during 1 hr. (Upper panel) locomotor and rearing activity of wt and *Bmal1-/-* mice on day1; (middle panel) relative locomotor activity (normalized to average distance covered on day1) and relative rearing activity (normalized to average rearing level on day1) of wt mice on days 1 and 2; (lower panel) relative locomotor and rearing activity  of *Bmal1-/-* mice on days 1 and 2 (* P<0.05).

The exploratory behavior of the wild type and *Bmal1-/-* mice in novel environment was tested in the open field paradigm (OF). For this, 3-months old male mice were placed in a bright-lit 50x50 inches square box and monitored for the pattern of their exploratory behavior for 1 hour with 5-min resolution. In order to assess intersessional habituation, animals were exposed to the same environment 24 hours later. Activity of *Bmal1-/-*  mice in novel environment was strikingly different from that of wild type mice. *Bmal1-/-* mice demonstrated significantly increased locomotion (horizontal activity) on day1 [F[1,5] = 19.21, P = 0.007] and day2 [F[1,5] = 27.36, P = 0.0004] (Figure 1, left panel).  Total distance traveled by *Bmal1-/-* animals during 1h on days 1 and 2 of the OF experiment was respectively 2.7-fold and 4.7-fold higher than that of the wild type mice. The pattern of activity in wild type and *Bmal1-/- *mice was also very different. As expected based on previous studies [1,16], on day1 the distance traveled by wild type mice during the first 15 min of the OF session was 3.1-fold higher than during the last 15 min [t-test P <0.0001]; on day2, it was 2.8-fold higher [t-test P <0.001]. Such a decrease with time on both first and second days of the experiment results from animals' habituation to a new environment within each OF session. When compared to day1, total activity of wild type mice on day2 was also significantly reduced [F[1,5] = 13.37, P = 0.015] (Figure 1 and 5, left), indicating that a long-term memory reflecting experience obtained on day1 has been formed [17]. In contrast, on day1 the difference in distance traveled by *Bmal1-/-* mice during the first 15 min of the OF session was only 1.6-fold higher than during the last 15 min [t-test P <0.01], whereas there was no difference on day2. Remarkably, the total distances traveled by *Bmal1-/-* mice during the first and the second days were virtually identical (Figure 5, lower left), and no statistically significant difference was detected between days 1 and 2 of the test [F[1,5] = 3.83, P = 0.107] (Figure1, left).

In the same experiments, vertical (rearing) activity was measured by registering the sequential crossing of beams up and down in vertical direction. Similar to the difference in horizontal activity, total rearing activity of *Bmal1-/-* mice was significantly increased compared to wild type animals (2.3-fold and 4.7-fold on days 1 [F[1,5] = 13.92, P = 0.014] and 2 [F[1,5] = 22.57, P = 0.00031], respectively (Figure 1, right panel).  On day2 wild type mice demonstrated 1.9-fold reduction in total rearing activity [F[1,5] = 24.34, P = 0.004], whereas no difference between the two days was detected in *Bmal1-/-* animals [F[1,5] = 0.37, P = 0.568] (Figure 5, lower right). In contrast to differences displayed in   horizontal activity, animals of both genotypes displayed similar rearing activity during the first 5 min of exposure to the new environment. After this, rearing activity of wild type animals gradually decreased, whereas in *Bmal1-/-* mice it increased and stayed elevated for the duration of testing (Figure 1, right). On day 2, the temporal pattern of rearing activity in animals of both genotypes did not differ much from the one displayed on day1. Taken together, these data indicate that deficiency in core circadian component, BMAL1, affects not only rhythmicity in locomotor activity, but other patterns of behavior as well. Specifically, when placed in a new environment, BMAL1-deficient mice display novelty-induced hyperactivity in locomotion and rearing behavior, and deficits in inter- and intrasessional habituation.

### BMAL1 is expressed in hippocampal and cortical neurons

**Figure 2.**
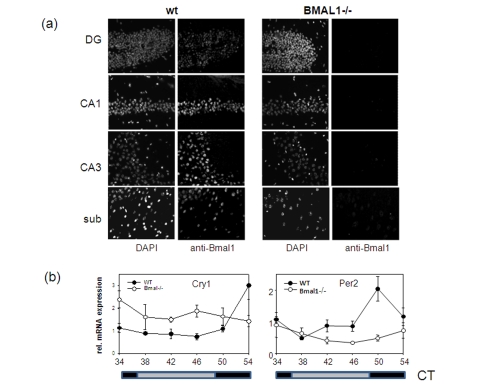
Expression of circadian proteins in brain structures. (**a**) Immunostaining of sagittal brain sections of wt and *Bmal-/-* mice with BMAL1 specific antibodies. Counterstaining with DAPI was used to detect nuclei. Pyramidal neurons of hippocampal areas CA1 and CA3, granular cells of the dentate gyrus and neurons of subiculum expressing BMAL1 are shown. (**b**) Circadian profile of *Cry1* and *Per2* mRNAs in the brain of wt (filled circles) and *Bmal1-/- *mice (open circles) as measured by real-time PCR. (* p<0.05).

BMAL1 is expressed in many different tissues and organs. Brain-specific *Bmal1* expression at the mRNA level has been demonstrated for the suprachiasmatic nucleus of the anterior hypothalamus (SCN),  the residence of the master circadian clock, as well as for other brain regions including the cortex and hippocampus formation [12,18]. To investigate the distribution of BMAL1 protein in the brain, we performed *in situ* immunofluorescent staining using BMAL1-specific antibody (Figure 2a). High expression of BMAL1 was detected in the pyramidal neurons of the hippocampus and in the neurons of the subiculum and enthorhinal cortex of wt mice. Neurons of the neocortex were also positive for BMAL1. Specificity of the signal was confirmed by the parallel staining of the brains of *Bmal1-/-* mice. Thus, BMAL1 protein is expressed in brain structures associated with memory formation.

### BMAL1 deficiency disrupts circadian expression of *mPer2* and *mCry1* genes in the brain

In complex with CLOCK (or its close tissue-specific homolog, NPAS2) BMAL1 controls rhythmic expression of target genes, and BMAL1 deficiency results in disruption of rhythmic pattern of gene expression in the SCN and liver [12]. We decided to investigate how the absence of BMAL1 will affect the expression of BMAL1 target genes in the brain. As demonstrated in Figure 2b, rhythmic pattern of expression of two core circadian genes, *mPer2* and *mCry1* in the brain of BMAL1-deficient animals was significantly impaired with the *mPer2* gene being mostly down-regulated and *mCry1* up-regulated. This pattern of expression was previously observed in the SCN and liver of *Bmal1*-/- mice and was attributed to the dual role of the BMAL1:CLOCK complex in transcription regulation [19]. Importantly, both PER2 and CRY1 were recently implicated in memory and learning [20,21].

### BMAL1 deficiency results in disruption of ROS homeostasis in the brain

Aging is associated with increased oxidative stress in many tissues, including the brain. Recently, oxidative stress and misbalance in reactive oxygen/nitrogen species (ROS/RNS) homeostasis was proposed as a mechanism for age-dependent changes in brain physiology, including decline in memory and learning [22]. Previously we have demonstrated that BMAL1 is directly involved in the regulation of ROS/RNS homeostasis, and that accelerated aging characteristic for  *Bmal1-/-* mice, at least in part, can be attributed to excessive production of ROS in some tissues of *Bmal1-/-* animals [4]. This prompted us to compare the levels of ROS in the brain of wild type and *Bmal1-/-* mice.

**Figure 3.**
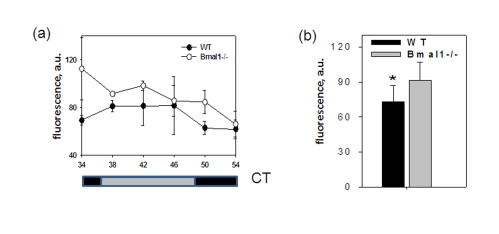
BMAL1 deficiency disrupts ROS homeostasis in the brain. (**a**) ROS level in the brain of wt and *Bmal1-/-* mice was detected in the indicated time points of the circadian cycle. (**b**) Average ROS level in the brain of wt and *Bmal1-/-* mice for 24h (* P<0.05).

To account for possible daily fluctuations, the level of ROS was measured in brains of wild type and *Bmal1-/-* mice collected throughout the day every 4 hrs. The level of ROS in the total brain extracts did not show any obvious circadian pattern; however, at each time point it was significantly higher in *Bmal1-/-* mice (except for the time of maximum level of ROS for the wild type (CT34)). As a result, in mutant mice the average daily levels of ROS in the brain were 20% higher [t-test P < 0.01] (Figure3b). Thus, BMAL1 deficiency results in excessive production of ROS and chronic oxidative stress in the brain, which may affect various brain-specific metabolic processes including memory formation.

### Deficiency of circadian proteins CLOCK and CRY1,2 differentially alters habituation and exploratory activity

The observed behavioral phenotype of *Bmal1-/-* mice may result from desynchronization of physiological activity of neurons due to disruption of the circadian oscillator. On the other hand, it may be unique to *Bmal1-/-* mice and result from disruption of the BMAL1-dependent control of tissue homeostasis. To discriminate between these two possible mechanisms in the regulation of hyperactivity and habituation, we studied the exploratory behavior of arrhythmic mice with disrupted activity of other circadian proteins.

We have chosen mice with the deficiency of the two *Cry* genes (*Cry1,2-/- *double knockout mice) and mice with the homozygous mutation of the BMAL1 transcription partner, CLOCK (*Clock/Clock *mutant mice). Previous work has shown that these two models, along with the *Bmal1-/-* model, may be approximated by two opposite functional states of the BMAL1:CLOCK transcription complex. Thus, functional deficiency in BMAL1 or CLOCK proteins results in the absence of transactivation of the target genes, while the absence of the CRY1,2 proteins cause constantly elevated expression of circadian target genes. All these mutants demonstrate disruption of rhythmic pattern of locomotor activity and at the gene expression level [12-14].

**Figure 4.**
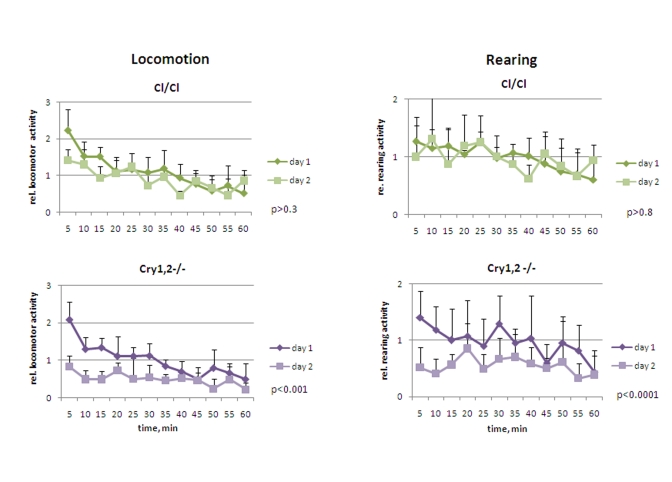
Open field analysis of exploratory activity and habituation of *Clock/Clock* and *Cry1,2-/-* mice. Relative locomotor and rearing activity of *Clock/Clock* and *Cry1,2-/-*  mice on days 1 and 2 (normalized to the average distance/activity level on day1) (* P<0.05).

**Figure 5.**
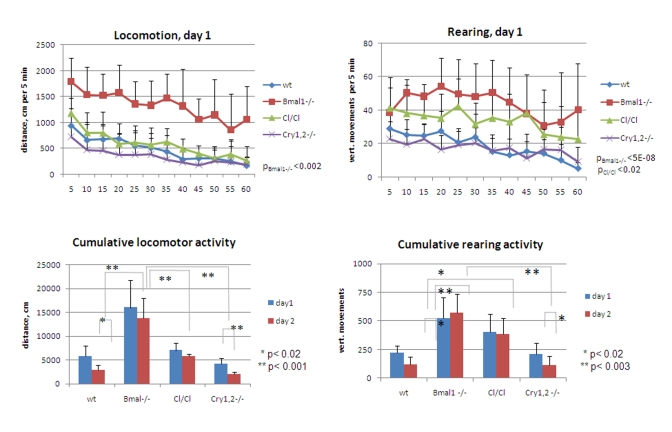
** Exploratory activity of wild type and circadian mutant mice.** (**Upper panel**) Locomotor and rearing activity measured in 5 min increments during 1 hr on day1 for wt (diamonds), *Bmal1-/-* (squares), *Clock/Clock* (triangles) and *Cry1,2-/-* (crosses) mice. Statistically significant difference with wt activity is shown as p values. Difference between wt, *Clock/Clock* and *Cry1,2-/-* locomotor activities, and between rearing activities of *Bmal1-/-* vs. *Clock/Clock* and wt vs. *Cry1,2-/-* is not statistically significant. (**Lower panel**) Cumulative traveled distance and rearing activity on days 1 and 2; For upper panels p values of statistically significant differences between wild type and circadian mutants are indicated. For lower panels * P<0.05.

*Cry1,2-/-* and *Clock/Clock* mice were placed in novel environment, similarly to experiments described for wild type and *Bmal1-/-* animals. In contrast to *Bmal1-/-* mice, they did not demonstrate hyperactivity in locomotion: the activity of *Clock/Clock* on day1 was indistinguishable from that of the wild type [F[1,5] = 1.77, P = 0.241], while *Cry1,2-/-* animals were even less active [F[1,5] = 3.23, P = 0.132] (Figure 4 and 5, left panels). Both *Clock/Clock* [F(11,55) = 13.12, P<0.001] and *Cry1,2-/-* [F(11,55) = 8.02, P<0.001] mice showed intrasessional habituation on day1 similar to that of the wild type mice: distance traveled during the last 15 minutes decreased more than 2 fold compared to the first 15 minutes. On day2, *Clock/Clock* mice demons-trated the level of locomotor activity indistinguishable [F[1,5] = 3.95, P = 0.103] from that on day1.  Thus, although *Clock/Clock* mice showed intrasessional habituation similar to wild type (both on days 1 and 2), there was no intersessional habituation (no significant difference between days 1 and 2). These data suggest that that *Clock/Clock* mutant mice have normal formation of the immediate memory of novel context and impaired long-term memory. Locomotor activity of *Cry1,2-/-* mice on day2 was significantly lower than on day1 (Figures 4 and 5, left) (2.0 folds, [F[1,5] = 27.19, P = 0.003]), suggesting that *Cry1,2-/-* demonstrate both intra- and intersessional habituation.

While the level of horizontal activity of *Clock/Clock* mutants was similar to the horizontal activity of wild type mice, *Clock/Clock* mutants demonstrated elevated rearing activity [F[1,5] = 7.65,  P = 0.04], which was intermediate between that of wild type and *Bmal1-/-* animals (Figure 4 and 5, right). In contrast with the case of locomotion (normal intarsessional habituation and no intersessional habituation), there was no difference in rearing activity of *Clock/Clock* mutants between day1 and day2 [F[1,5] = 0.44, P = 0.538], and only insignifi-cant decrease in rearing between the first and the last 15 min of the experiment on both days (T-test P = 0.1 and P = 0.6, respectively). Rearing behavior of *Cry1,2-/-* mice on day1 was similar to wt (with a tendency to be lower) (T-test=0.6) (Figure 5, right); however, rearing activity of *Cry1,2-/-* on day2 constantly remained at the habituated level and was significantly lower than wt [F[1,5] = 48.63, P<0.001] (Figure 4, right).

Taken together, these results demonstrate a correlation between the level of activity and memory formation on one hand and transcription status of the BMAL1:CLOCK complex on the other.

### Deficiency in activity of the core circadian proteins BMAL1, CLOCK or CRY1,2 results in different behavioral patterns in the open field

Cumulative data on locomotor and rearing activities showing significant differences between the animals of all tested genotypes are summarized in Figure 5. Thus, total distance traveled by *Bmal1-/-* mice on both days greatly exceeded the distance traveled by the wild type [day1 fold 2.8, t-test P <0.01; day2 fold 4.7, P <0.001], *Clock/Clock* [day1 fold 2.3, t-test P <0.01; day2 fold 2.4, P <0.01], or *Cry1,2-/-* [day1 fold 3.9, t-test P <0.001; day2 fold 6.9, P <0.001] animals (Figure5, left panels). *Cry1,2-/-* mice demonstrated the lowest level of horizontal activity, while the locomotion of the *Clock/ Clock* animals was comparable to that of the wild type. Remarkably, the horizontal activity of *Bmal1-/-* and *Clock/Clock* mutants remained the same on both days of the experiment, whereas wild type and *Cry1,2-/-* animals demonstrated significant reduction in activity on day2 [wt fold 2.0 t-test P <0.02; *Cry1,2-/-* fold 2.1, P <0.01] (Figure 5, lower left). Rearing activity of *Bmal1-/-* and *Clock/Clock* animals was elevated compared to wt [*Bmal1-/-* day1 fold 2.3, t-test P <0.01, day2 fold 4.7, P <0.001; *Clock/Clock* day1 fold 1.8, P <0.01, day2 fold 3.2, P <0.001] and showed no differences between the two sessions. In contrast, wild type and *Cry1,2-/-* mice showed low rearing activity and robust intra- and intersessional habituation  (Figure 5, right panels).

**Figure 6.**
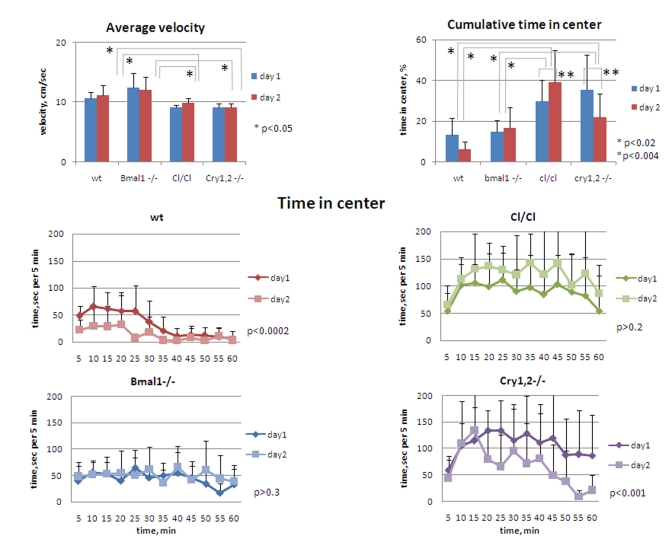
** Circadian mutant mice do not demonstrate anxiety phenotype.** (**Upper panel, left**) Average horizontal velocity for all genotypes measured during 1h on days 1 and 2.  Average velocity does not significantly differ between wt and *Bmal1-/-* animals; slight (~15%) but statistically significant decrease in average velocity is detected for *Clock/Clock* and *Cry1,2-/-* mice compared with wt and *Bmal1-/-*.  (**Upper panel, right**) Cumulative time spent in the center for all genotypes measured during 1 hr on days 1 and 2. (**Lower panel**) Time spent in the center square of the open field arena on days 1 and 2 by wt, *Clock/Clock, Bmal1-/-,* and *Cry1,2-/-* mice. *P<0.05.

### Circadian mutants demonstrate normal or decreased anxiety levels in the open field

The increase/decrease in the distance traveled by different circadian mutants may result from either differences in time spent in motion vs. rest time, or from the differences in the velocity among the genotypes. However, an average velocity of animals, calculated based on horizontal distance and time in horizontal motion, was mostly uniform in animals of all genotypes, except for *Clock* and *Cry1,2-/-*  mice that showed slight (~15%) but statistically significant reduction of speed [t-test P <0.05] (Figure 6, upper left). This suggests that *Bmal1-/-* and *Clock/Clock* mice were in  fact longer in motion, whereas *Cry1,2-/-* were less in motion on both days when compared to wild type, rendering a hyperactivity phenotype for *Bmal1-/-* and *Clock/Clock*animals.

High locomotor and rearing activity and deficit of contextual habituation often correlate with elevated level of anxiety, which can be accessed by the amount of time spent in the center of the OF (a risk-taking behavior) [23]. To evaluate the level of anxiety in animals of all four circadian genotypes, we compared the time they spent in the central zone of the OF (Figure 6). On day1 of the experiment, *Bmal1-/-* and wild type mice spent about 14% of the time in the central zone, while the corresponding time in *Cry1,2-/-* and *Clock/Clock* mice was more than two-fold higher (37% and 30% respectively [t-test P <0.01]) (Figure 6, upper right).  The differences between the wild type and *Bmal1-/-,* and between *Clock/Clock* and *Cry1,2-/-* animals were not significant. Compared to day1, the amount of time spent in the center on day2 was not changed in *Bmal1-/-* , whereas in wild type and *Cry1,2-/-* mice it was decreased two-fold [t-test P <0.01]. Interestingly, cumulative center time of *Clock/Clock* mice on day2 showed even a tendency for increase but did not reach statistical significance. Thus, none of the tested circadian mutants displayed a pro-anxiety phenotype in the open field paradigm. On the contrary, *Clock/Clock* and *Cry1,2-/-* demonstrated opposite, more risk-taking behavior. A decrease in center time on day2 observed in wild type and *Cry1,2-/-* mice correlates with a decrease in total locomotor and vertical exploratory activity (indicative of habituation).  *Bmal1-/-* again did not demonstrate any difference in performance between days 1 and  2, while *Clock/Clock* mutant mice demonstrated an increase in time spent in the center. Interestingly, mice of different genotypes had different patterns of the risk-taking behavior. Wild type mice "took the risk" of short raids in the middle of the brightly-lit arena during the first 30 min and then moved mainly along the walls or sit in one of the corners (Figure 6, middle left). *Bmal1-/-* mice continued to move across the center during the entire session (Figure 6, lower left). Both *Cry1,2-/-* and *Clock/Clock* mice had an increase in time spent in the center after first 10 min, during which they had prolonged periods of sitting in the middle of the arena - a very unlikely behavior for wt mice (Figure 6, middle and lower right). These data clearly demonstrate the lack of correlation between the locomotor/rearing activity and time spent in the center of the open field in different circadian mutants. Thus, hyperactive *Bmal1-/-* demonstrated "normal" level of anxiety, while both hyperactive *Clock/Cl*ock and hypoactive *Cry1,2-/-* had decreased level of anxiety. Therefore, hyperactivity and deficit of contextual habituation of *Bmal1-/-* and *Clock/Clock* cannot be explained by the increase in the level of anxiety in these mice.

## Discussion

Decline in mental performance, including deficits in memory formation, learning and adaptation to novelty are hallmarks of aging. At the same time, it is well documented that the activity of the circadian clock decreases with age [24]. Reciprocal relationships between the decline in the circadian clock activity and deterioration in mental performance are currently a subject for active discussions [25]. In this study we propose that the activity of the circadian proteins is important for adaptation to novelty, which is one of the aspects of daily interactions between an organism and its changing environment. Our results demonstrate that habituation to novelty is differentially altered in mice with a deficiency/mutation of the core circadian genes *Bmal1*, *Clock*, or *Cry1* and *Cry2 * and correlates with the transcription activity status of the BMAL1:CLOCK: [CRY1,2]  complex.

Exploration behavior is thought to be induced by a novelty detected by the hippocampus which works as a comparator of the stored spatial "maps" - memory of visited places - and perception of an unknown space. As the animal acquires information of the new space, a novel spatial map is generated and exploratory behavior ceases (which is referred to as intrasession habituation in the open field paradigm, and depends on working memory). When placed in the same environment on consequent days, mice demonstrate significantly reduced exploratory activity, which is interpreted as a sign of acquiring of long-term memory about the place (intersessional habituation) [1-3,17]. Thus, habituation is thought to depend on short- and long-term memory [26-28]. Deficiency in the intrasession habituation of *Bmal1-/-* mice is indicative for working memory impairments. Recently, circadian modulation of short-term memory was shown in Drosophila [29] and humans [30].  Severe deficiency in the intersession habituation demonstrated by *Bmal1-/-* and *Clock/Clock* mice both in locomotion and rearing suggests that *Bmal1-/-* and *Clock/Clock* mice lack memory of the previous day experience and allows speculating that transcription activity of the BMAL1:CLOCK complex is necessary for the LTM formation, which requires *de novo* synthesis of both RNA and protein [31].  Importantly, LTM was shown to depend on time of the day for LTM acquisition/retrieval [32], which may reflect daily fluctuations in BMAL1:CLOCK transcription activity.  Facilitation of both intra- and intersession habituation demonstrated by *Cry1,2-/-* mice further strengthens the role of BMAL1, CLOCK and CRY1,2-associated transactivation and transrepression of gene expression in memory function. Interestingly, although *Cry1,2-/-* mice exhibit a deficit in time-place learning, which was attributed to disrupted time-keeping system, no deficits were observed in learning abilities of *Cry1,2-/-* mice in several not-time associated learning tasks [21]. Our results suggest that LTM and/or STM formation in these animals is facilitated; however, more specific learning/memory tests are necessary to dissect various types of memory influenced by CRY1,2 as well as other circadian proteins. These observations and several recent reports indicate a close connection between the activity of the circadian system and memory formation. Circadian cycling was recently proposed as a mechanism for the proper memory consolidation [33], probably through the circadian oscillation of MAP kinase activity reported in the mouse hippocampus [34]. Circadian modulation of memory formation has been shown for different model organisms such as Aplysia, Drosophila, zebrafish and rodents; a growing body of evidence implicates the circadian regulation of learning and cognitive performance in humans [11]. More data on specific roles of individual circadian proteins in different forms of memory are accumulated from studies of mice with deficiencies in these proteins. Thus, mice deficient in NPAS2 have impaired cued and contextual fear memory [35]. *Per2-/-* mice demonstrate impairment in trace fear memory, but not in cued fear memory [20]. *Per1-/-* mice exhibit spatial learning deficits in the radial arm maze [18].

Exposure to a novel environment is mildly stressful and inherently arousing experience for mice [1-3]. Failure of the *Bmal1-/- *mice to habituate within a single session could also be attributed to their inability to cope with the novelty-induced stress resulting from functional disruption of one or several brain modulatory systems [2], which might be the cause of hyperactivity in both locomotion and rearing in these mice. Indeed, the circadian clock is involved in control of the rate-limiting enzyme in the biosynthesis of dopamine - tyrosine hydrolase (TH, also known as monooxygenase) expression; TH expression is reduced in *Per1*-deficient mice [9], whereas it is greatly elevated in *Clock/Clock* mutants [8]. Significant hyperactivity in the open field was reported for several transgenic mice with disturbed dopamine regulation [36]. Interestingly, recently was shown that modulation of the hippocampus-dependent memory by attention is dopamine-mediated [37]. Further study on dopamine level and bioavailability in circadian mutants will help to determine whether the observed changes in the activity of circadian mutants occur through dopamine-dependent or independent mechanisms. On the other hand, hyperactivity is often associated with elevated anxiety [38]; however, the anxiety level of *Bmal1-/-* mice did not significantly differ from the wild animals judging by the time spent in the center of the arena. Importantly, hyperactivity of *Bmal1-/- *mice was associated with novelty, because average home cage activity does not differ between wt and *Bmal1-/-* animals [12]. In contrast, *Clock/Clock* mutant mice exhibited the pattern of horizontal activity similar to wild type; however, their rearing activity was almost two-fold higher compared with wt. Horizontal locomotion correlates with cognitive component of exploratory behavior, while rearing behavior is considered to reflect motivational component [28]; together with the fact that *Clock/Clock* mutants spent in the center of the open field arena twice more time than wt animals, these data suggest that *Clock/Clock* mice demonstrate enhanced activity-based arousal and reduced anxiety, which is in good agreement with observations made by [39]. In sharp contrast to *Bmal1-/-* and *Clock/Clock* mutant mice, *Cry1,2-/-* mice were less active in the open field experiments; at the same time, time spent in the center of the open field arena was almost three-fold higher in *Cry1,2-/-* mice when compared to wild type and *Bmal1-/-*, and was comparable with that of the *Clock/Clock* mutants - the pattern which can be interpreted as a sign of greatly reduced anxiety in these animals. These observations reinforce the previously reported data on the involvement of the circadian proteins in the regulation of mood [8,9].

ROS/RNS are important regulators of cellular signaling; any misbalance can be critical for brain physiology and affect various mental functions, [40-42], therefore, their production and detoxification are tightly controlled by the system of ROS/RNS-generating and antioxidant enzymes. Chronic oxidative stress in an aging brain is one of the main reasons for age-associated mental decline [22]. Here we show that BMAL1 deficiency significantly disturbs the normal ROS level in the brain. Thus, BMAL1-dependent control of ROS can be one of the potential mechanisms of the observed behavioral changes of the circadian mutants. Indeed, as already has been mentioned above, the circadian oscillation of MAP kinase activity in the hippocampus is critical for memory formation, although the mechanisms of cyclic activity of MAPK are unclear [34]. ROS are critical regulator of MAP kinase activation and MAP kinase signaling pathway [43], thus, the observed circadian oscillation of ROS level in the brain can be at least partially responsible for the oscillation of MAPK activity.

Using experimental settings within the open field paradigm, which embraces the established behavioral tests for exploration and adaptation to novelty in rodents, we have found that activity of the core circadian clock proteins BMAL1, CLOCK and CRY1,2 is necessary for the regulation of exploratory behavior in mice. Opposite phenotypes of *Bmal1-/-* and *Clock/Clock* mutant mice on one hand and *Cry1,2-/-* on the other suggest that the changes in the novelty-induced behavior in these animals are not the result of the general disruption of the circadian clock, but rather indicate that individual protein components of  the molecular clock play non-identical roles in habituation. Therefore, the exploratory performance depends on the mutual balance of activities of these proteins, while the general regulation of these activities by the circadian clock warrants the optimization of the performance. It is well documented that aging affects the circadian system [24]; here we suggest that aging also affects the mutual balance between circadian proteins, which in turn affects mental performance. Our results suggest the involvement of the circadian proteins in fundamental processes of memory formation, and encourage further investigations into the role of the circadian proteins in memory, learning behavior and age-associated mental decline.

## Experimental procedures

Animals. *Bmal1-/-*mice were obtained from Dr. C. Bradfield (University of Wisconsin)  [12], *Clock* mutant mice were obtained from Dr. J. Takahashi (Northwestern University) [13], and *Cry1,2-/-*knockout mice were obtained from Dr. A. Sancar (University of North Carolina at Chapel Hill) [14]; details of target gene knockout strategies and animal generations can be found in the above cited publications. All mutants were backcrossed to C57BL/6J inbred strain (The Jackson Laboratory, Bar Harbor,   ME, USA) for 12 generations. Wild type and *Bmal1-/-* mice were generated by breeding of *Bmal1+/-* males with *Bmal1+/-* females. *Clock* mutants were generated by breeding of *Clock/Clock* males with *Clock/wt* females, *Cry1,2-/-* were generated by breeding of *Cry1,2-/-* males with *Cry1+/-, Cry2-/-* females. Wild type mice generated as a result of *Bmal1+/-* breeding were used as a control for all experiments (since after 10 backcross generations the line is 99% genetically identical to the recipient strain, mutants (backcrossed to C57BL/6J for 12 generations) and wild type were considered as congenic with C57BL/6J background).  For all experiments wild type and mutant mice were randomly picked from several independent litters. Animals were maintained on a 12:12 light:dark cycle with lights on at 7:00 am, on regular diet. For tissue collection animals were transferred to constant darkness and tissue samples were collected with 4 hour intervals beginning after 34 hours of exposure to DD, immediately frozen on dry ice and stored at - 80°C. All animal studies were conducted in accordance with the regulations of the Committee on Animal Care and Use at Cleveland State University and Roswell Park Cancer Institute.

Open field exploration. A mouse was placed in the bright-lit 50x50 inches Plexiglas square box, and the activity of the animal was monitored with 16x16 photobeam activity system (San Diego Instruments). Animal activity was recorded every 5 minutes during 1 hour on the day1 and day2 (24h later), and analyzed using Open Field Software. All experiments were performed with 3-4 month old male mice between 11 am and 4 pm, at least 6 animals of each genotype were analyzed.

RNA isolation and real-time PCR analysis. Total RNA was isolated from the brain with TriZol reagent (Invitrogen) according to the manufacturer's protocol. RNA quantitation was performed using TaqMan real-time RT-PCR, relative mRNA abundance was calculated using the comparative delta-Ct method with GAPDH mRNA as standard. Procedure and primer sequence was previously described [19].

Immunohistochemical analysis. Frozen brain coronal sections (10 μm) were fixed with 4% PFA dissolved in PBS (pH 7.5) for 10 min, permeabilized with 0.5% Triton X-100 for 5 min. The sections were incubated with primary anti-BMAL1 antibodies raised in guinea pig  followed by incubation with donkey anti-guinea pig secondary antibody labeled with DyLight488 (Jackson ImmunoResearch laboratories), incubated for 1 min with 4'-6-Diamidino-2-phenylindole (DAPI, 300nM, Invitrogen), mounted under cover slips using Fluoromount G  media (SouthernBiotech). The slides were kept in the dark at +4^o^C until use. Microphotographs were taken with the aid of Leica DMR upright microscope equipped with Princeton Instruments MicroMax 5 MHz-cooled CCD camera and ImagePro software.

ROS analysis. ROS levels were determined in tissue extracts using ROS sensitive fluorescent dye as described elsewhere [4]. Briefly, brain was immediately frozen on dry ice and stored at -70^o^ C until analysis. After mixing with 10 volumes of homogenization buffer and normalizing by protein content, brain extracts were mixed with dichlorodihydrofluorescein (DCF) in homogenization buffer and incubated in the dark at 37^ o^ C for 30 min. Fluorescence at 495/535nm was measured using Victor2 Wallac microplate reader (Perkin Elmer). At least 3 animals were used for analysis for every time point and genotype.

Statistical analysis. Six male mice of each genotype were used for all experiments. Data are shown as mean + standard deviation. SigmaStat software package was used for analysis. Effects of genotype (circadian mutants versus wild type) and novel/familiar environment (day1 versus day2) on behavioral variables collected in open field experiments were tested for significance with Two Way Repeated Measures ANOVA. Bonferoni t-test was used for all pairwise multiple comparison procedures. Unpaired Student's t-test was used for comparison of total activities between day1 and day2 for the same genotype or the same day for different genotypes. Unpaired Student's t-test was used for comparison of between genotype variations in relative gene expression and ROS level at different time points. P<0.05 was considered as statistically significant.

## References

[1] Cerbone A, Sadile AG (1994). Behavioral habituation to spatial novelty: interference and noninterference studies. Neurosci Biobehav Rev.

[2] Leussis MP, Bolivar VJ (2006). Habituation in rodents: a review of behavior, neurobiology, and genetics. Neurosci Biobehav Rev,.

[3] Rankin CH, Abrams T, Barry RJ, Bhatnagar S, Clayton DF, Colombo J, Coppola G, Geyer MA, Glanzman DL, Marsland S, McSweeney FK, Wilson DA, Wu CF, Thompson RF (2009). Habituation revisited: an updated and revised description of the behavioral characteristics of habituation. Neurobiol Learn Mem.

[4] Kondratov RV, Kondratova AA, Gorbacheva VY, Vykhovanets OV, Antoch MP (2006). Early aging and age-related pathologies in mice deficient in BMAL1, the core componentof the circadian clock. Genes Dev.

[5] Kondratov RV, Gorbacheva VY, Antoch MP (2007). The role of mammalian circadian proteins in normal physiology and genotoxic stress responses. Curr Top Dev Biol.

[6] Franken P, Dijk DJ (2009). Circadian clock genes and sleep homeostasis. Eur J Neurosci.

[7] Abarca C, Albrecht U, Spanagel R (2002). Cocaine sensitization and reward are under the influence of circadian genes and rhythm. Proc Natl Acad Sci U S A.

[8] McClung CA, Sidiropoulou K, Vitaterna M, Takahashi JS, White FJ, Cooper DC, Nestler EJ (2005). Regulation of dopaminergic transmission and cocaine reward by the Clock gene. Proc Natl Acad Sci U S A.

[9] Hampp G, Ripperger JA, Houben T, Schmutz I, Blex C, Perreau-Lenz S, Brunk I, Spanagel R, Ahnert-Hilger G, Meijer JH, Albrecht U (2008). Regulation of monoamine oxidase A by circadian-clock components implies clock influence on mood. Curr Biol.

[10] Roybal K, Theobold D, Graham A, DiNieri JA, Russo SJ, Krishnan V, Chakravarty S, Peevey J, Oehrlein N, Birnbaum S, Vitaterna MH, Orsulak P, Takahashi JS, Nestler EJ, Carlezon WA Jr, McClung CA (2007). Mania-like behavior induced by disruption of CLOCK. Proc Natl Acad Sci U S A.

[11] Gerstner JR, Lyons LC, Wright KP Jr, Loh DH, Rawashdeh O, Eckel-Mahan KL, Roman GW (2009). Cycling behavior and memory formation. J Neurosci.

[12] Bunger MK, Wilsbacher LD, Moran SM, Clendenin C, Radcliffe LA, Hogenesch JB, Simon MC, Takahashi JS, Bradfield CA (2000). Mop3 is an essential component of the master circadian pacemaker in mammals. Cell.

[13] Antoch MP, Song EJ, Chang AM, Vitaterna MH, Zhao Y, Wilsbacher LD, Sangoram AM, King DP, Pinto LH, Takahashi JS (1997). Functional identification of the mouse circadian Clock gene by transgenic BAC rescue. Cell.

[14] Vitaterna MH, Selby CP, Todo T, Niwa H, Thompson C, Fruechte EM, Hitomi K, Thresher RJ, Ishikawa T, Miyazaki J, Takahashi JS, Sancar A (1999). Differential regulation of mammalian period genes and circadian rhythmicity by cryptochromes 1 and 2. Proc Natl Acad Sci U S A.

[15] Harms E, Kivimae S, Young MW, Saez L (2004). Posttranscriptional and posttranslational regulation of clock genes. J Biol Rhythms.

[16] Kalueff AV, Jensen CL, Murphy DL (2007). Locomotory patterns, spatiotemporal organization of exploration and spatial memory in serotonin transporter knockout mice. Brain Res.

[17] Wilson DA, Linster C (2008). Neurobiology of a simple memory. J Neurophysiol.

[18] Jilg A, Lesny S, Peruzki N, Schwegler H, Selbach O, Dehghani F, Stehle JH (2010). Temporal dynamics of mouse hippocampal clock gene expression support memory processing. Hippocampus.

[19] Kondratov RV, Shamanna RK, Kondratova AA, Gorbacheva VY, Antoch MP (2006). Dual role of the CLOCK/BMAL1 circadian complex in transcriptional regulation. FASEB J.

[20] Wang LM, Dragich JM, Kudo T, Odom IH, Welsh DK, O'Dell TJ, Colwell CS (2009). Expression of the circadian clock gene Period2 in the hippocampus: possible implications for synaptic plasticity and learned behaviour. ASN Neuro.

[21] Van der Zee EA, Havekes R, Barf RP, Hut RA, Nijholt IM, Jacobs EH, Gerkema MP (2008). Circadian time-place learning in mice depends on Cry genes. Curr Biol.

[22] Bishop NA, Lu T, Yankner BA (2010). Neural mechanisms of ageing and cognitive decline. Nature.

[23] Stansfield KH, Kirstein CL (2007). Chronic cocaine or ethanol exposure during adolescence alters novelty-related behaviors in adulthood. Pharmacol Biochem Behav.

[24] Hofman MA, Swaab DF (2006). Living by the clock: the circadian pacemaker in older people. Ageing Res Rev.

[25] Reddy AB, O'Neill JS (2009). Healthy clocks, healthy body, healthy mind. Trends Cell Biol.

[26] Crusio WE (2001). Genetic dissection of mouse exploratory behaviour. Behav Brain Res.

[27] O'Keefe J (1993). Hippocampus, theta, and spatial memory. Curr Opin Neurobiol.

[28] Lever C, Burton S, O'Keefe J (2006). Rearing on hind legs, environmental novelty, and the hippocampal formation. Rev Neurosci.

[29] Lyons LC, Roman G (2009). Circadian modulation of short-term memory in Drosophila. Learn Mem.

[30] Vandewalle G, Archer SN, Wuillaume C, Balteau E, Degueldre C, Luxen A, Maquet P, Dijk DJ (2009). Functional magnetic resonance imaging-assessed brain responses during an executive task depend on interaction of sleep homeostasis, circadian phase, and PER3 genotype. J Neurosci.

[31] Costa-Mattioli M, Sossin WS, Klann E, Sonenberg N (2009). Translational control of long-lasting synaptic plasticity and memory. Neuron.

[32] Chaudhury D, Colwell CS (2002). Circadian modulation of learning and memory in fear-conditioned mice. Behav Brain Res.

[33] Roth TL, Sweatt JD (2008). Rhythms of memory. Nat Neurosci.

[34] Eckel-Mahan KL, Phan T, Han S, Wang H, Chan GC, Scheiner ZS, Storm DR (2008). Circadian oscillation of hippocampal MAPK activity and cAmp: implications for memory persistence. Nat Neurosci.

[35] Garcia JA, Zhang D, Estill SJ, Michnoff C, Rutter J, Reick M, Scott K, Diaz-Arrastia R, McKnight SL (2000). Impaired cued and contextual memory in NPAS2-deficient mice. Science.

[36] Gainetdinov RR, Wetsel WC, Jones SR, Levin ED, Jaber M, Caron MG (1999). Role of serotonin in the paradoxical calming effect of psychostimulants on hyperactivity. Science.

[37] Muzzio IA, Kentros C, Kandel E (2009). What is remembered? Role of attention on the encoding and retrieval of hippocampal representations. J Physiol.

[38] Belzung C, Griebel G (2001). Measuring normal and pathological anxiety-like behaviour in mice: a review. Behav Brain Res.

[39] Easton A, Arbuzova J, Turek FW (2003). The circadian Clock mutation increases exploratory activity and escape-seeking behavior. Genes Brain Behav.

[40] Gahtan E, Auerbach JM, Groner Y, Segal M (1998). Reversible impairment of long-term potentiation in transgenic Cu/Zn-SOD mice. Eur J Neurosci.

[41] Hidalgo C, Carrasco MA, Munoz P, Nunez MT (2007). A role for reactive oxygen/nitrogen species and iron on neuronal synaptic plasticity. Antioxid Redox Signal.

[42] Serrano F, Klann E (2004). Reactive oxygen species and synaptic plasticity in the aging hippocampus. Ageing Res Rev.

[43] Torres M (2003). Mitogen-activated protein kinase pathways in redox signaling. Front Biosci.

